# Combinatorial treatment with statins and niclosamide prevents CRC dissemination by unhinging the MACC1-β-catenin-S100A4 axis of metastasis

**DOI:** 10.1038/s41388-022-02407-6

**Published:** 2022-08-25

**Authors:** Benedikt Kortüm, Harikrishnan Radhakrishnan, Fabian Zincke, Christoph Sachse, Susen Burock, Ulrich Keilholz, Mathias Dahlmann, Wolfgang Walther, Gunnar Dittmar, Dennis Kobelt, Ulrike Stein

**Affiliations:** 1 Experimental and Clinical Research Center, Charité—Universitätsmedizin Berlin and Max-Delbrück-Center for Molecular Medicine in the Helmholtz Association, Berlin, Germany; 2 German Cancer Consortium (DKTK), Heidelberg, Germany; 3 NMI-TT Pharmaservices, Berlin, Germany; 4 Charité University Hospital Berlin Centre 10 Charite Comprehensive Cancer Center, Berlin, Germany; 5 Max-Delbrück-Center for Molecular Medicine in the Helmholtz Association, Berlin, Germany

**Keywords:** Prognostic markers, Colorectal cancer, Metastasis, Targeted therapies, Tumour biomarkers

## Abstract

Colorectal cancer (CRC) is the second-most common malignant disease worldwide, and metastasis is the main culprit of CRC-related death. Metachronous metastases remain to be an unpredictable, unpreventable, and fatal complication, and tracing the molecular chain of events that lead to metastasis would provide mechanistically linked biomarkers for the maintenance of remission in CRC patients after curative treatment. We hypothesized, that Metastasis-associated in colorectal cancer-1 (MACC1) induces a secretory phenotype to enforce metastasis in a paracrine manner, and found, that the cell-free culture medium of MACC1-expressing CRC cells induces migration. Stable isotope labeling by amino acids in cell culture mass spectrometry (SILAC-MS) of the medium revealed, that S100A4 is significantly enriched in the MACC1-specific secretome. Remarkably, both biomarkers correlate in expression data of independent cohorts as well as within CRC tumor sections. Furthermore, combined elevated transcript levels of the metastasis genes MACC1 and S100A4 in primary tumors and in blood plasma robustly identifies CRC patients at high risk for poor metastasis-free (MFS) and overall survival (OS). Mechanistically, MACC1 strengthens the interaction of β-catenin with TCF4, thus inducing S100A4 synthesis transcriptionally, resulting in elevated secretion to enforce cell motility and metastasis. In cell motility assays, S100A4 was indispensable for MACC1-induced migration, as shown via knock-out and pharmacological inhibition of S100A4. The direct transcriptional and functional relationship of MACC1 and S100A4 was probed by combined targeting with repositioned drugs. In fact, the MACC1-β-catenin-S100A4 axis by statins (MACC1) and niclosamide (S100A4) synergized in inhibiting cancer cell motility in vitro and metastasis in vivo. The MACC1-β-catenin-S100A4 signaling axis is causal for CRC metastasis. Selectively repositioned drugs synergize in restricting MACC1/S100A4-driven metastasis with cross-entity potential.

## Introduction

CRC is second in tumor incidence and cancer lethality worldwide. Metastatic spread accounts for over 90% of deaths in CRC patients, despite improvements of surgical and adjuvant treatments [1–3]. About 30% of CRC patients without metastases at diagnosis (UICC stage I-III) are expected to develop metachronous metastases in distant organs. Identifying these high risk CRC patients for targeted therapy to prevent relapse remains an unmet clinical need [4].

Upregulation of MACC1 in primary tumors is linked to metachronous metastasis and, independently of the stage of disease, predicts poor metastasis-free survival in CRC and more than 20 other solid tumor entities [5, 6].

MACC1 itself has been identified as an inducer of CRC metastasis through several distinct mechanism [5]. The proto-oncogene MET is induced transcriptionally by MACC1, leading to stabilized HGF/MET signaling [5, 7]. Structurally, the MACC1 molecule serves as a scaffold for MEK1, leading to prolonged and potentiated ERK1 activation [8]. Furthermore, MACC1 promotes the dynamin-dependent restoration of the growth factor receptor EGFR in the cell membrane [9, 10]. These capabilities fuel various cancer hallmarks, such as enhanced survival, proliferation, motility, and metastasis of cancer [6, 11–15].

We have reported previously that the widely used statin drugs effectively downregulate MACC1 expression in CRC cells, accompanied with potent anti-migratory and metastasis-preventive effects in vivo [16–18].

CRC is frequently initiated by hyperactivated Wnt/β-catenin signaling due to loss of APC function or gain of β-catenin function. This allows β-catenin to accumulate in the cytoplasm and to translocate into the nucleus, where it activates Wnt target genes that sustain cell proliferation, motility, and stemness [19, 20]. Additionally, a variety of kinases stabilize β-catenin, resulting in enforcement of its transcriptional effects on cancer promoting target genes [21–23]. The metastasis driver S100A4, one of the calcium binding S100 proteins, is regulated transcriptionally by Wnt/β-catenin signaling [24]. Restriction of β-catenin-dependent expression of S100A4 results in reduced cell motility and prevents metastasis formation in vivo [25, 26]. Intracellularly, S100A4 increases cell motility by interacting with the cytoskeletal proteins F-actin and non-muscle myosin-IIa [27, 28]. In the extracellular space, secreted S100A4 enhances MEK-ERK signaling via its binding to RAGE [29]. It was also reported to shape the premetastatic niche in vivo, and to crucially determine organotropism of solid cancer metastasis [30–32]. As a versatile enforcer of cancer progression and metastasis, S100A4 is a stage-independent predictor for metachronous metastasis in CRC and other solid tumor entities [24, 33].

We were able to demonstrate that circulating transcripts of MACC1 and S100A4 are detectable in blood of cancer patients and serve as reliable biomarkers for OS and MFS. Combined elevation of MACC1 and S100A4 identified patients with the highest risk for unfavorable prognosis in CRC, gastric, and ovarian cancer [34–36]. These findings led us to investigate a potential novel functional link between the strong prognostic markers MACC1 and S100A4. We discovered that through β-catenin signaling, MACC1 directly induces S100A4 expression and secretion, and further mediates MACC1 pro-migratory effect on cancer cells through S100A4 as the enforcing molecule. Here we report on a novel MACC1-β-catenin-S100A4 axis and demonstrate that, based on this rationale, combined transcriptional inhibition of MACC1 and S100A4 restricts cancer cell motility and metastasis in CRC with seminal cross entity potential for cancer therapy.

## Results

### MACC1 expression stimulates cell motility via secreted proteins

**Fig. 1:**
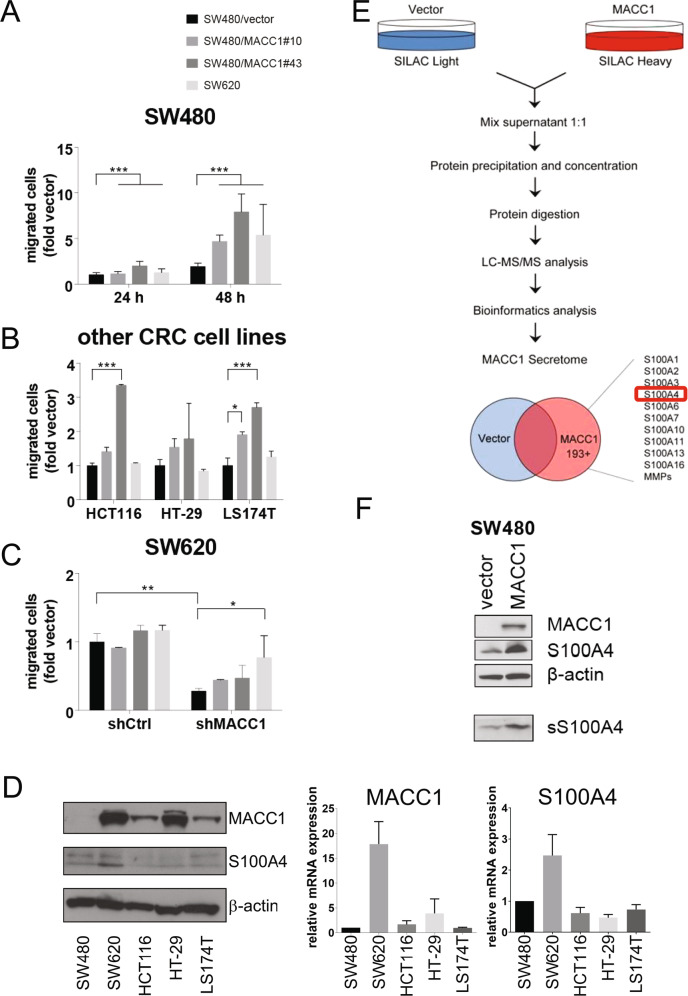
The MACC1 secretome induces CRC cell migration via S100A4. Culture medium of MACC1-overexpressing cells stimulated migration of SW480 cells (**A**), and in HCT116, HT-29 and LS174T (**B**). The same culture supernatants rescued migration of SW620/shMACC1 that was diminished after depletion of MACC1 (**C**). Baseline protein (left) and mRNA (right) expression of MACC1 and S100A4 in the human colon carcinoma cell lines SW480, SW620, HCT116, HT-29 and LS174T (**D**). In a SILAC analysis of cell culture medium of SW480/vector and SW480/MACC1 cells, S100 proteins were secreted de novo in MACC1-overexpressing cells (**E**). Western blot from cell culture supernatant (sample volume proportional to respective cell count at time of medium harvest) confirmed increased presence of soluble S100A4 (sS100A4) in the secretome of SW480/MACC1 cells (**F**). Numeric results shown means ± SEM of 3 independent experiments, test for significance with ANOVA and Tukey correction for multiple testing.

We hypothesized that MACC1-overexpressing tumor cells stimulate cell migration by secretion of pro-metastatic factors. Cell-free culture supernatants from SW480/vector cells, two independent SW480/MACC1 clones (#10, #43) expressing MACC1 ectopically, or SW620 cells, which express high intrinsic MACC1 levels, were added to the CRC cell line SW480, which intrinsically expresses minimal amounts of MACC1. After 48 hours of exposure to MACC1-conditioned medium, the motility of SW480 cells was assessed in Boyden chamber assays. The supernatant of MACC1-overexpressing cells increased the transwell migration capacity of SW480 cells, while the control culture exposed to SW480/vector medium demonstrated poor motility (Fig. 1A). This was verified in three additional CRC cells lines (Fig. 1B). The medium of SW620 cells showed only minor efficacy to induce cell migration in SW480, HCT116, HT-29 and LS174T. Knockdown of MACC1 in SW620 cells (SW620/shMACC1) led to a reduction of migration compared to SW620/shCtrl. None of the conditioned media yielded significant effects on the motility of SW620/shCtrl cells. Intriguingly, SW480/MACC1-conditioned medium failed to rescue migration in SW620/shMACC1 cells, while medium from wildtype SW620 significantly increased migration in SW620/shMACC1 cells (Fig. 1C). MACC1 baseline expression is lowest in SW480 and highest in SW620 cells. HCT116, HT-29 and LS174T feature moderate expression of MACC1. S100A4 is stably expressed in all cell lines, with the highest expression in SW620 cells (Fig. 1D). We carried out a mass-spectrometric SILAC analysis to identify the active principles of the MACC1-specific secretome of SW480/MACC1 cells (vs. SW480/vector cells) (Fig. 1E). Soluble S100A4 was amongst the de novo secreted proteins in MACC1-conditioned cell medium. The increase of S100A4 secretion by SW480/MACC1 cells was confirmed by direct western blotting from cell culture medium (Fig. 1F).

### Combination of MACC1 and S100A4 robustly identifies high risk CRC patients

**Fig. 2:**
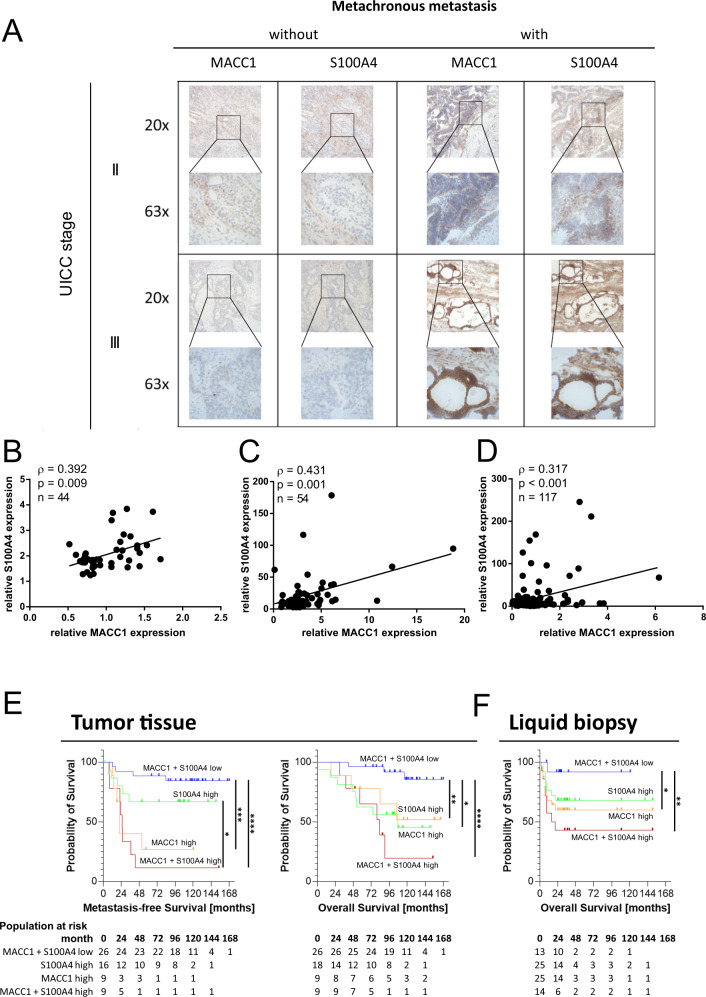
MACC1 and S100A4 robustly identify high risk CRC patients. IHC of MACC1 and S100A4 in each 2 tumors of non-metastasized and metachronously metastasized primary tumors confirms overexpression of both biomarkers in CRC that yielded metachronous metastases well after surgical removal of the primary tumor (**A**). MACC1 and S100A4 expressions correlate in CRC tumors. Gene expression levels were analyzed in three additional cohorts of 44, 54 and 117 CRC tumors, respectively. Co-expression was examined with Spearman correlation, and we found a positive correlation of MACC1 and S100A4 expression levels in all datasets (**B**–**D**). Kaplan–Meier analysis for MFS and OS of patients based on the MACC1-S100A4 combination panel, test for significance with log rank (Mantel-Cox) test. Combined overexpression of MACC1 and S100A4 was associated with dismal MFS and OS in primary CRC tumors (**E**), and high levels of MACC1 and S100A4 mRNA transcripts detected in liquid biopsies (preoperative blood samples) predicted poor OS (**F**). Significant intergroup differences are indicated with asterisks, where applicable.

To test whether MACC1-dependent expression of S100A4 is a clinically prevalent phenomenon in CRC, we detected both markers by IHC in representative tumor sections from a previously published cohort. Non-metastasized primary CRC tumors presented a low immunoreactivity for MACC1 and S100A4. Primary tumors associated with metachronous metastases strongly expressed both MACC1 and S100A4 (Fig. 2A). To further validate the co-expression of MACC1 and S100A4 in CRC samples, we correlated both genes in three independent CRC patient cohorts [37–39]. In all cohorts MACC1 and S100A4 showed a significant positive correlation (Spearman-ρ = 0.392, *p* = 0.009; Spearman-ρ = 0.431, *p* = 0.001; Spearman-ρ = 0.317, *p* < 0.001, respectively) (Fig. 2B–D). Since both markers have been reported individually as stage-independent prognostic biomarkers across multiple cancer entities, we assessed the combined prognostic value of MACC1 and S100A4 for MFS and OS. We quantified mRNA levels of MACC1 and S100A4 in primary tumors from a cohort of 60 CRC patients (UICC stage I-III), and of a plasma sample set from a previously published cohort of CRC patients, with RT-qPCR. Based on these data we established respective cut-off values for the primary endpoints “metachronous metastasis” and “death” [5, 34]. Within the primary tumor cohort, the subgroup “MACC1 and S100A4 high” experienced the shortest MFS, while the subgroup “MACC1 and S100A4 low” showed the longest MFS (median follow up: 167.2 months; median MFS of 69 and 131 months, respectively; *p* < 0.0001; Fig. 2E). Similarly, the subgroup “MACC1 and S100A4 high” experienced the shortest OS, while the subgroup “MACC1 and S100A4 low” showed the longest OS (*p* = 0.0025; Fig. 2E). We extended the follow-up on patient survival in the liquid biopsy cohort, and found that also here, combined increased expression of MACC1 and S100A4 was associated with poor OS, while low expression of both markers was found in the subgroup with favorable OS (median follow up: 27.5 months; mean OS of 70 and 111 months, respectively; *p* = 0.007; Fig. 2F).

These findings strongly suggest the existence of a hitherto unknown mechanistic link between MACC1 and S100A4 in metastasis.

### MACC1 induces S100A4

**Fig. 3:**
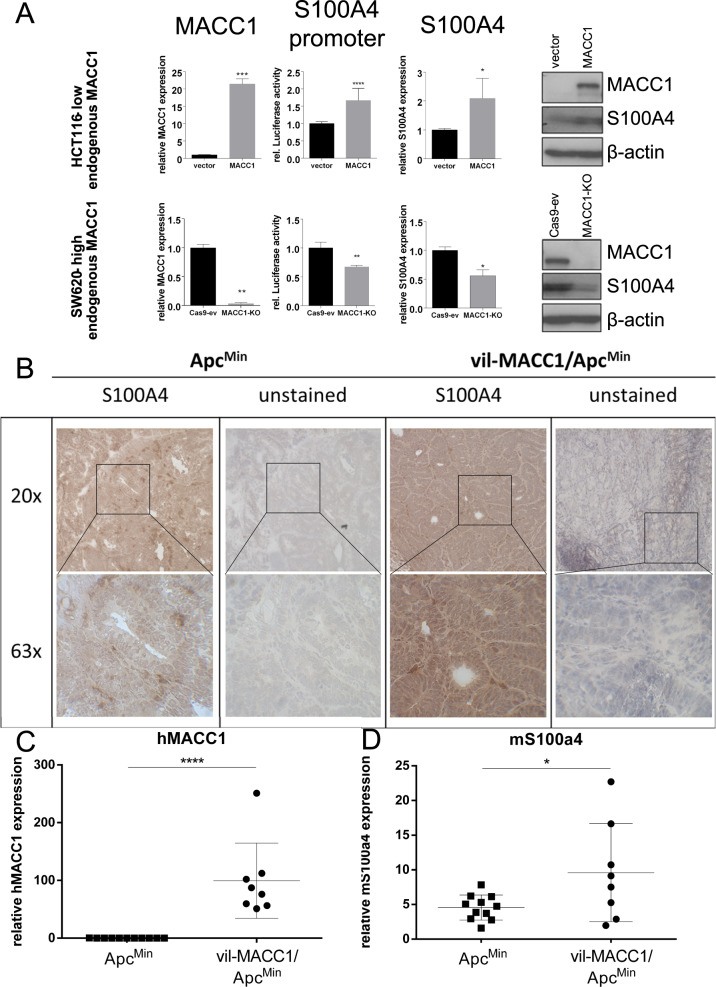
MACC1 promotes S100A4 in vitro and in vivo. Ectopic MACC1 in HCT116 increased the activity of a S100A4 promoter-driven luciferase reporter. Concomitantly, S100A4 was increased in mRNA and protein levels (**A** top). Knockout of MACC1 in SW620 was followed by reduced S100A4-promoter-driven luciferase as well as decreased S100A4 mRNA and protein expression (**A** bottom). IHC of S100A4 in tumors of Apc^Min^ and vil-MACC1/Apc^Min^ mice confirms overexpression of S100A4 in MACC1-overexpressing littermates (**B**). In tumors of vil-MACC1/Apc^Min^ that overexpress human MACC1 (hMACC1, **C**), mouse-intrinsic S100a4 (mS100a4) was upregulated at RNA level (**D**).

The observation of correlated MACC1 and S100A4 expression and activity prompted the examination of a causal link between these initially unrelated mediators of metastasis. Regulation of S100A4 expression by MACC1 was examined with a luciferase-based reporter for S100A4 promoter activity, and at S100A4 mRNA and protein level. In HCT116 cells with endogenously low MACC1 expression, ectopic MACC1 overexpression induced luciferase activity in TOP-flash assays and S100A4-promoter reporter plasmids. This is complemented by increased S100A4 mRNA and protein levels (Fig. 3A, top row). Conversely, CRISPR-Cas9-mediated knockout of MACC1 in SW620 cells with endogenous high MACC1 expression led to decreased TOP-flash and S100A4-promoter activity and concomitant downregulation of S100A4 mRNA and protein (Fig. 3A, bottom row). Additionally, we examined the Apc^Min^ mouse model of APC/β-catenin-dependent intestinal tumors for susceptibility of such effects of MACC1 in vivo by means of IHC and RT-qPCR. In randomly selected tumors of our previously published vil-MACC1/Apc^Min^ mouse model [12], mouse-intrinsic S100a4 (mS100a4) was significantly increased at protein (Fig. 3B) and mRNA level (Fig. 3C) compared to Apc^Min^ littermates, that lack villin-dependent MACC1 overexpression (Fig. 3D).

### MACC1 employs S100A4 through β-catenin signaling to drive cancer cell motility

**Fig. 4:**
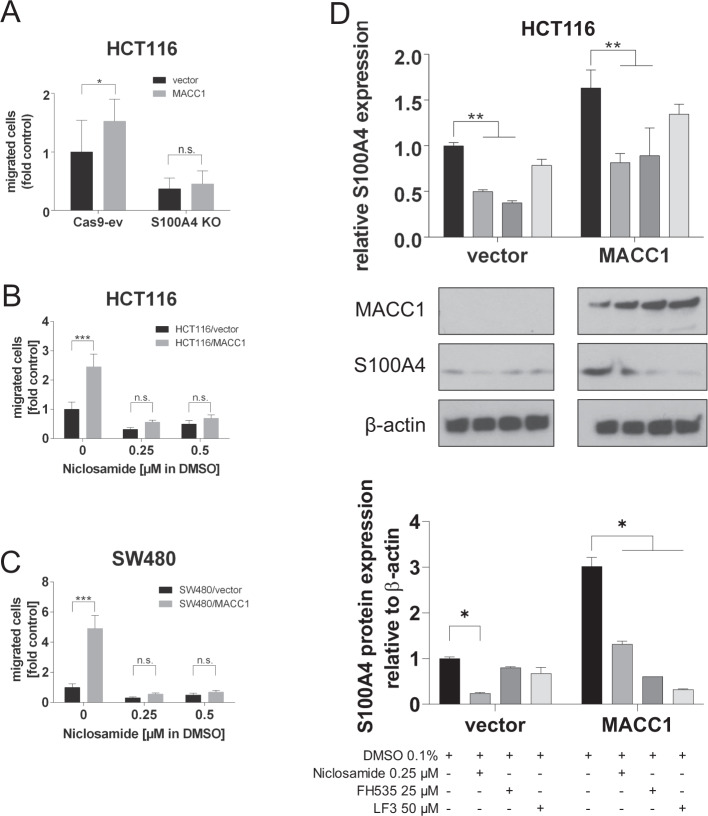
MACC1 employs S100A4 through β-catenin signaling to drive cancer cell motility. MACC1-specific cell motility depends on S100A4. Overexpression of MACC1 in HCT116 cells induced transwell migration, but not in S100A4-KO counterparts (**A**). MACC1-induced cell migration in HCT116 and SW480 cells, and this effect was reverted by niclosamide, a transcriptional inhibitor of S100A4 (**B**, **C**). MACC1 increased S100A4 in presence of DMSO, and three independent β-catenin inhibitors (niclosamide, FH535 and LF3) largely reversed this upregulation on mRNA and protein level to expression levels of HCT116/vector cells (**D**). Numeric results shown as means ± SEM of 3 independent experiments, test for significance with Student’s *t*-test, or ANOVA and Tukey correction for multiple testing.

To investigate whether MACC1-induced cell motility is directly mediated by S100A4, we studied the effect of MACC1 overexpression in S100A4-depleted cells. S100A4 was knocked out in HCT116 cells using CRISPR-Cas9 technology, while cells transfected with Cas9 and without sgRNA served as empty vector control (Cas9-ev). MACC1 was overexpressed in both S100A4 competent (Cas9-ev) and in S100A4 deficient (S100A4-KO) HCT116 cells. MACC1 increased transwell migration capability in Cas9-ev cells. No statistically significant change in the S100A4-KO cells’ migratory ability was seen, which demonstrated overall reduced motility (Fig. 4A). We inhibited S100A4 with its transcriptional inhibitor niclosamide and found significant reduction of transwell migration in HCT116/MACC1 and SW480/MACC1 cells, but not in HCT116/vector or SW480/vector cells, respectively (Fig. 4B, C). Prochlorpromazine, an FDA-approved phenothiazine drug and potent inhibitor of S100A4 interaction with myosin, proved similarly effective in migration inhibition (Fig. S1A) [40, 41]. Using knockout as well as pharmacological inhibition of S100A4 we show that MACC1-induced cell motility is largely dependent on S100A4 expression and function. S100A4 is a β-catenin signaling target gene itself [24]. Therefore, we tested whether β-catenin inhibitors would intercept the MACC1-dependent upregulation of S100A4. Indeed, niclosamide [25] and two other β-catenin inhibitors FH535 [42] and LF3 [43] hindered the upregulation of S100A4 in MACC1-overexpressing cells at mRNA and protein level in HCT116 cells (Fig. 4D). In SW480 cells, niclosamide dose-dependently downregulated the expression of S100A4 in both MACC1-overexpressing cells and in vector-transfected cells (Fig. S1B). These findings support that MACC1 regulates S100A4 expression through β-catenin/TCF4 signaling, creating the MACC1-β-catenin-S100A4 signaling axis.

### MACC1 binds to β-catenin and stabilizes its interaction with TCF4

**Fig. 5:**
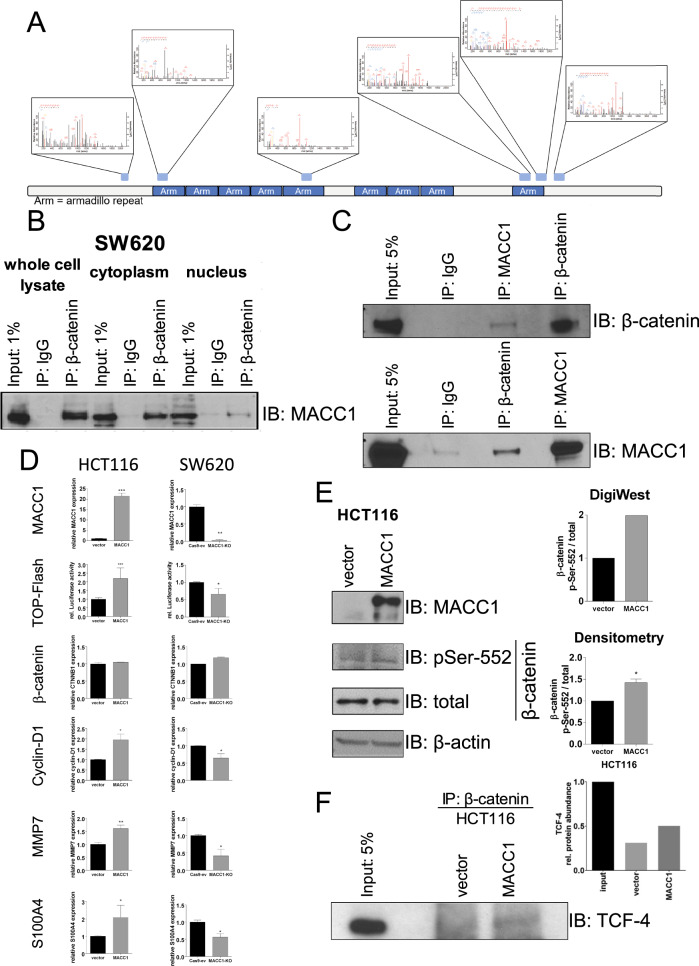
MACC1 interacts with β-catenin and induces its transcriptional activity via post-translational modification. In a mass-spectrometry-based analysis of the MACC1 interactome in SW620 cells, several peptides interacting with MACC1 mapped to β-catenin, suggesting a direct PPI (**A**). Co-IP experiments on whole-cell lysates, cytoplasmic and nuclear protein of SW620 confirmed direct interaction between MACC1 and β-catenin (**B**). This finding was recapitulated by co-incubating recombinant MACC1 and β-catenin proteins in cell-free Co-IP assays (**C**). Impact of MACC1 overexpression or MACC1 knock-out is shown on TCF-reporter activity and mRNA expression of β-catenin and the β-catenin/TCF target genes Cyclin-D1, MMP7 and S100A4 in HCT116 and SW620 cells. In HCT116 cells MACC1 overexpression increases TCF reporter activity and TCF target gene expression, while β-catenin gene expression itself is unaffected. In SW620 cells MACC1 knockout decreases TCF reporter activity and TCF target gene expression, whereas β-catenin gene expression is also unaffected (**D**). In a DigiWest experiment, MACC1-overexpressing HCT116 cells demonstrated increased phosphorylation of p-Ser-552-β-catenin, confirmed in semiquantitative western blots, while total β-catenin protein was not altered (**E**). Comparative immunoprecipitation of β-catenin protein showed increased binding of TCF4 in HCT116/MACC1 cells, also shown by densitometry (**F**). Boxplots show means ± SEM of 3 independent experiments, test for significance with Student’s *t*-test.

To comprehensively describe MACC1 interactions within β-catenin signaling we employed mass-spectrometry on co-immunoprecipitated MACC1 interaction partners and determined the MACC1-interactome. Intriguingly, we found β-catenin as an interaction partner of MACC1 (Fig. 5A). Co-Immunoprecipitation (Co-IP) experiments confirmed this protein-protein interaction (PPI) in whole cell lysates as well as in cytoplasmic and nuclear fractions (Fig. 5B). To probe whether this PPI is of direct nature or through other proteins, recombinant human MACC1 and β-catenin were co-incubated in an equimolar ratio in lysis buffer to carry out cell-free Co-IP experiments. Indeed, IP of MACC1 did precipitate β-catenin protein and vice versa, indicating that MACC1 and β-catenin maintain a direct PPI (Fig. 5C). We speculated that MACC1 interacts with β-catenin to mediate its posttranslational stabilization and transcriptional activity. While MACC1 overexpression increased the activity of the TOP-flash reporter for β-catenin/TCF4 signaling, mRNA levels of β-catenin where not affected. However, its target genes S100A4, cyclin-D1 and MMP7 were upregulated by MACC1 (Fig. 5D). Using DigiWest technology, we detected increased phosphorylation of β-catenin on Ser-552 (Fig. 5E), a post-translational modification (PTM) linked to stabilization and enhanced binding to TCF4, which was confirmed by western blot (Fig. 5F). In summary, MACC1 facilitates the phosphorylation of β-catenin at Ser-552 and improves β-catenin/TCF4 interaction to enhance the expression of β-catenin/TCF4 target genes including S100A4.

### Combined transcriptional inhibition of MACC1 and S100A4 synergizes in restricting cancer cell motility and metastasis

We have shown the direct and causal functional link of MACC1 and S100A4 driving cell motility. To translate this finding into a novel therapeutic strategy we combined the transcriptional inhibition of both MACC1 and S100A4 by small molecules. For this, we investigated potential synergisms between inhibitors of MACC1 and S100A4 and applied atorvastatin or further members of the statin family fluvastatin and lovastatin (targeting MACC1 expression) and niclosamide (targeting S100A4 expression) as monotherapy or in combination. We analyzed the combination for reduced proliferation and motility in wound healing assays in HCT116 cells, which express both MACC1 and S100A4, at three different drug concentrations [17, 25].

**Fig. 6:**
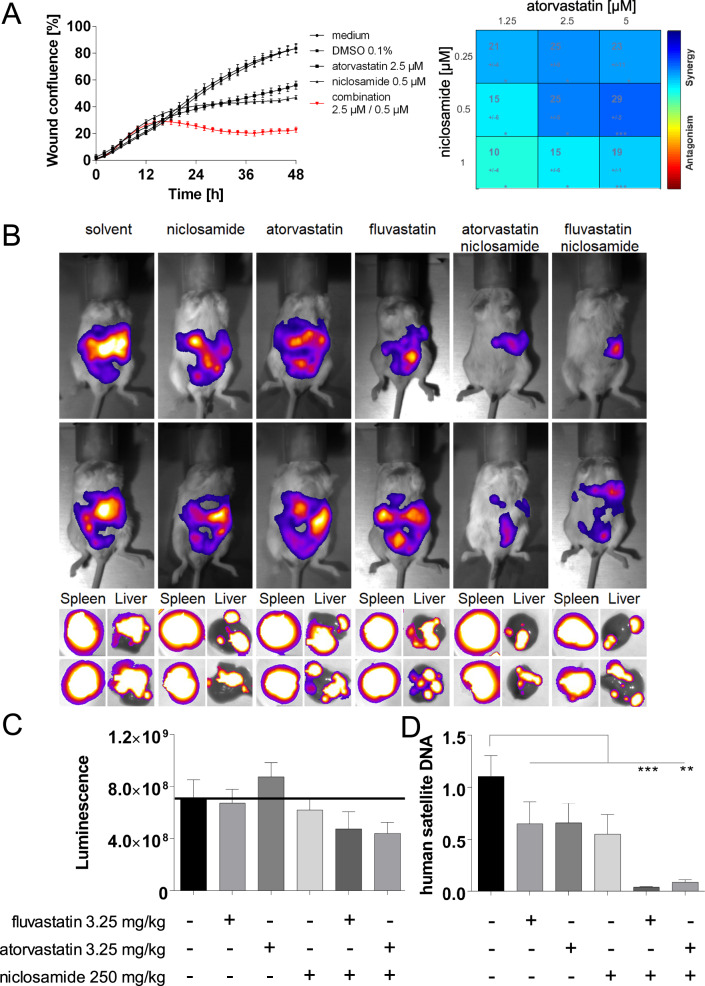
Statins and niclosamide synergize in suppression of CRC cell wound healing and metastasis. HCT116 human colorectal cancer cells with endogenous MACC1 and S100A4 expression were treated with three concentrations (1.25, 2.5 and 5 µM) of atorvastatin and niclosamide (0.25, 0.5 and 1 µM) alone and in combinations thereof (**A**). After drug application in vitro wound healing was monitored for 48 h using the IncuCyte live cell imaging system. Atorvastatin and niclosamide were able to reduce wound closure compared to control cells. Combining a statin with niclosamide increased this effect synergistically. Results are shown as means ± SEM of at least 3 experiments. Treating xenografted SCID beige mice (*n* = 60, 10 animals per group) with human equivalent doses of either atorvastatin (3.25 mg/kg, 20 mg per patient per day), fluvastatin (3.25 mg/kg, 20 mg per patient per day) or niclosamide (250 mg/day, 1.5 g per patient per day) alone reduced metastasis formation in the liver. Confirming the in vitro results, the combination of the tested drugs, each statin with niclosamide, was superior to single drug treatment (**B**, **D**). While visualization of xenografted cells by luminescence did not show significant reductions just before mouse killing (**C**), human cell load in the murine liver was quantifiably reduced in human satellite DNA specific qPCR (**D**). Numeric results are shown as mean ± SEM, and level of significance indicated as per ANOVA and Dunnett’s correction for multiple testing.

Single treatments with atorvastatin (2.5 µM) and niclosamide (0.5 µM) significantly reduced wound closure. Combination of atorvastatin and niclosamide demonstrated a synergistic inhibitory effect, compared to the respective single treatments (Fig. 6A, Supplementary movie 1). This effect was shown to be dose-dependent (Fig. 6A, right panel).

Next, we tested two additional statins (fluvastatin and lovastatin) in combination with niclosamide. These statins also show a synergistic effect in combination with niclosamide (Fig. S4A, B, supplementary movies 2, 3). In summary, statins inhibit cellular motility synergistically with niclosamide even at lower concentrations (Figs. 5A and S3A, B). Synergy analyses indicated the synergism to be highest for 2.5 µM of a statin in combination with 0.5 µM niclosamide.

To test the combinatorial MACC1/S100A4 inhibition for metastasis intervention in vivo, SCID beige mice were intrasplenically xenotransplanted with stably luciferase overexpressing HCT116 cells. The tested statins and niclosamide were orally administered at human equivalent dosages [44]. Drug administration and monitoring of tumor growth and metastasis started 5 days after cell inoculation. The mice were treated with atorvastatin, fluvastatin or niclosamide alone, or with combinations thereof. A control group received solvent only. Solvent-treated mice developed extensive liver metastases over time as monitored by bioluminescence. Metastasis formation was quantified by human satellite DNA load in the livers. Single treatments with atorvastatin, fluvastatin or niclosamide significantly reduced liver metastasis by 41%, 40% and 50%, respectively (Fig. 6B–D). More importantly, the combinatorial treatment with atorvastatin and niclosamide restricted liver metastases, reflected by reduced bioluminescence. Human satellite DNA in mouse livers was reduced by 96% and 92%, respectively, in mice under atorvastatin/niclosamide and fluvastatin/niclosamide treatment (Fig. 6D). Synergistic effects are dosage dependent. Given at 100% human equivalent concentrations, all treatment regimens strongly reduced in vivo bioluminescence from mouse liver metastases, and combinatorial treatments were not superior to monotherapies (Fig. S4A). At 12.5% human equivalent dosage, neither treatment affected splenohepatic metastasis of CRC xenografts (Fig. S4B). Neither drug regimen exerted any discernible toxicity reflected in comparable relative body weight curves (Fig. S4C). We additionally stained human CRC cells within the murine liver via IHC of human CK19. In livers of mice receiving monotherapies diminished hCK19 immunoreactivity suggested a reduction of micrometastases compared to solvent-treated animals (1: solvent, 2: niclosamide, 3: fluvastatin, 4: atorvastatin). This effect was more pronounced in livers of combination treated animals (5: niclosamide + fluvastatin, 6: niclosamide + atorvastatin) (Fig. S4E). This is supportive for the efficacy of multiple hit intervention of the MACC1-β-catenin-S100A4 axis to reduce metastasis. The results were confirmed in a second CRC in vivo model. SW620 cells with high endogenous expression of MACC1 and S100A4 were intrasplenically xenografted in SCID beige mice. Treatment with the highest human equivalent dose of 328 mg/kg (human dose 2 g per day and patient) niclosamide or 13 mg/kg (human dose 80 mg per day and patient) of each statin alone failed to reduced liver metastasis. However, combination of niclosamide and one of the two tested statins showed a strong reduction of liver metastasis for both approaches (Fig. S5A, B).

## Discussion

This is the first report to functionally link two metastasis-associated genes, MACC1 and S100A4, and to elucidate their cooperation as inducer (MACC1) and enforcer (S100A4) of CRC cell motility and metastasis in one functionally shared MACC1-β-catenin-S100A4 axis.

Mechanistically, MACC1 induces S100A4 overexpression on a transcriptional level, in that it interacts with β-catenin, induces its posttranslational stabilization and enhances its interaction with TCF4 [24].

Importantly, we found S100A4 to be instrumental in MACC1 mediated cell motility. MACC1 promoted transwell migration only in S100A4-proficient cells and failed to do so in S100A4-depleted cells and under pharmacological inhibition of S100A4 transcription or activity. MACC1 facilitated an activating PTM of β-catenin, which has been linked to enhanced transcriptional activity of its target genes [45–47]. MACC1 did not increase β-catenin expression, neither at mRNA nor protein level. However, the effect on Ser-552 phosphorylation might be crucial to permit MACC1 mediated hyperactivation of β-catenin signaling [48–50]. We speculate that MACC1, which lacks sequences suggestive of ATP-binding or kinase activity [6], rather acts as an adapter protein and recruits kinases to β-catenin. Further studies should probe for joined nuclear shuttling of MACC1 and β-catenin and examine the modulation of β-catenin-dependent transcription complexes. These findings have identified MACC1 as one decisive piece in the complex puzzle of intracellular signal transduction of cancer progression and metastasis.

The MACC1-β-catenin-S100A4 axis is functional in clinical cases of CRC, in which co-expression of MACC1 and S100A4 in tumors as well as patient blood hallmark high-risk CRC patients. We stratified CRC patients according to MACC1- and S100A4-transcript levels in primary tumors and in blood serum, respectively. The combined MACC1/S100A4 biomarker robustly identified CRC patients at high risk for poor MFS by tumor RNA analysis and predicted poor OS from liquid biopsy analyses. In sections of locally advanced, non-metastasized CRC (UICC stages II and III), MACC1 and S100A4 expression coincided, and discriminated metachronously metastasized cases from non-metastasized cases. Furthermore, both biomarkers correlated positively in three independent CRC cohorts.

The molecular background of a given tumor dictates its phenotype and aggressiveness and thus predicts patient survival. In light of this, several studies have identified molecular subtypes of CRC with distinct clinicopathological features impacting therapy response and survival [51, 52]. Personalized cancer medicine seeks to maximize therapeutic efficacy and minimize escape of tumor cells by resistance mechanisms by targeting the individual molecular makeup of a given tumor. We have previously described an association of MACC1 in KRAS-driven CRC, where MACC1 overexpression combined with KRAS G13 mutation conferred poor MFS [53]. Conversely, absent MACC1 expression in mismatch-repair deficient CRC identifies low-risk patients who would not benefit from, and could be spared adjuvant chemotherapy [54]. Independently, our research as well as other studies confirm the prognostic value of S100A4 in CRC and other malignancies [33, 55, 56]. In this report we demonstrate that rationally combining two mechanistically linked biomarkers indeed improve prediction of CRC metastasis and cancer survival.

Our observation, that MACC1 and S100A4 form a signaling axis through β-catenin might add to characterization of molecular cancer subtypes with high metastatic potential and novel intervention points for improved anti-metastatic therapy. It seems likely that MACC1 and S100A4 also cooperate in other cancer entities, as the two biomarkers were described for improved prognosis of ovarian or gastric cancer, which underlines its considerable cross-entity potential [35, 36].

To test whether our hypothesis translates into clinical practice, we targeted the MACC1-β-catenin-S100A4 axis by combining transcriptional inhibitors of MACC1 (statins) and S100A4 (niclosamide) for cell motility and metastasis inhibition. We showed, that in combinatorial use, statins and niclosamide synergise in inhibiting CRC cell motility and invasion in vitro and metastatic dissemination in vivo. Metastasis remains the main challenge in the management of CRC patients and means of targeted metastasis intervention/inhibition or prevention is highly sought after. We demonstrate here that repositioning of statins and niclosamide, which are already in clinical use, could complement current therapy regimens for CRC [57, 58]. Long-term intake of statins, a mainstay of hypercholesterolemia management, has been associated with reduced cancer incidence in a variety of entities [59, 60]. The long term effects of niclosamide in cancer patients are currently tested in a clinical trial [61].

Here, we linked the metastasis inducer MACC1 to the metastasis enforcer S100A4 in a signaling axis through β-catenin, which promotes CRC progression and metastasis. Consequently, we simultaneously targeted inducer MACC1 and enforcer S100A4 within this axis of metastasis by combining clinically established drugs and generated efficient restriction of cancer cell motility and metastasis formation. These findings support the therapeutic value of our strategy with high translational potential, which warrants exploration in clinical trials for novel personalized, anti-metastatic therapies.

## Materials and methods

### Clinical samples

Tumor samples were obtained from 60 patients diagnosed with CRC of UICC stages I to III (no distant metastasis at time of diagnosis) undergoing R0 (no microscopic tumor residue) resections prior to tumor-specific adjuvant treatment. All patients had no history of hereditary colorectal cancer and did not have additional malignancies of any entity. Metachronous metastases developed in 23 patients (median follow-up: 167.2 months), 37 patients did not metastasize. The CRC cohort is identical with the cohort used in Stein et al. [5].

Blood samples were obtained from an independent cohort of 49 patients presenting at the Robert-Rössle-Clinic, Charité—Universitätsmedizin Berlin, with newly diagnosed CRC. Blood was taken on the day of diagnosis, prior to any surgical or adjuvant therapy, except of 16 patients diagnosed with locally advanced rectal cancer, who received neoadjuvant radio-chemotherapy of the primary tumor 3 to 6 days before blood was drawn. In these patients, no difference in circulating MACC1 transcript levels was detected in comparison to untreated rectal cancer patients. Presence of secondary malignancies in patient history or during follow-up was an exclusion criterion. The patient cohort is identical with the cohort studied by Stein, 2012 [34]. The follow-up period of survival was extended to a median of 806 days after primary diagnosis, followed by re-evaluation of prognosis with the Kaplan Meier estimator.

### Cell culture, functional assays, and drug treatment

The CRC cell lines HCT116, SW480 and SW620 were maintained with RPMI or DMEM supplemented with 10% FBS in a humidified incubator at 37 °C and 5% CO_2_. Overexpression of MACC1 was achieved by lentiviral transduction of HCT116 cells with a MACC1-GFP construct (vector = GFP) and transfection of SW480 with a pcDNA3.1-MACC1-V5-His vector (vector = pcDNA3.1-V5-His). To knockout MACC1 in SW620 cells with CRISPR-Cas9 technology, predesigned plasmids encoding Cas9, puromycin resistance and sgRNA (Applied StemCell Inc., Milpitas CA, USA) were co-transfected. Following selection with 4 µg/ml puromycin for 48 h, viable cells were single-cell sorted into 96-well plates. Expanding clones were tested for MACC1 expression by western blotting. The genomic locus of MACC1 was sequenced to verify indel mutations in MACC1-deficient clones (Fig. S2, Table S1).

The migratory capacity of CRC cells was assessed in transwell migration assays. Briefly, 1 × 10^6^ cells were plated into 6-well plates. Complete medium was replaced by serum-free medium in the presence of inhibitors or DMSO, when indicated, and left overnight. 5 × 10^4^ cells were seeded into the pre-soaked transwell inserts of 96-well transwell migration plates (Corning Inc., Corning NY, USA). After 18 h at 37 °C, migrated cells were collected with Trypsin-EDTA and quantified with CellTiter Glo (Promega, Madison WI, USA). Data are accumulated from at least three independent biological replicates and technical quadruplicates.

Wound healing was measured in an IncuCyte ZOOM instrument (EssenBiosciences, Ann Arbor MI, USA). 1 × 10^5^ cells in 100 µl RPMI supplemented with 10% FCS were seeded into ImageLock 96-well plates (EssenBiosciences). After 6 h incubation to allow formation of monolayers, wounds were created with a WoundMaker tool (EssenBiosciences) and the wells were washed with PBS to remove floating cells. The plates were filled with complete medium, DMSO or inhibitors, when indicated, and wound closure was monitored continuously every 2 h. Analyses were performed by using the IncuCyte ZOOM 2016B software after generating confluence and wound masks with teaching image sets [62].

LF3, FH535, atorvastatin, fluvastatin, lovastatin, PCP (Selleckchem LLC, Houston TX, USA), and niclosamide (SigmaAldrich, St. Louis MO, USA) stocks were dissolved freshly for each experiment in DMSO (SigmaAldrich). The drugs were applied at concentrations indicated, while DMSO concentration was maintained at 0.1% in all treatments.

### Secretome analysis

Cells were grown in DMEM SILAC media supplemented with heavy lysine and arginine (^15^N_2_^13^C_6_ Lys, ^15^N_4_^13^C_6_ Arg). Secreted proteins were collected in the cell culture supernatant. The proteins were concentrated using a 30 kDa molecular filter cartridge (Millipore).

### Xenografting and in vivo treatment

3 × 10^5^ (HCT116/Luc) or 1 × 10^6^ (SW620) cells in 30 µl PBS were injected into the parenchyma of the spleen of 6-week-old female SCID bg/bg mice (*n* = 60). After cell inoculation the mice were randomly assigned to 6 groups. Mice were treated 3 days after transplantation p.o. daily with 10 ml/kg solvent solution (10% Kolliphor EL (SigmaAldrich; 0.9% NaCl), 1.5–13 mg/kg atorvastatin or fluvastatin, or 164–328 mg/kg niclosamide in solvent). These amounts correspond to the human doses of 10 -80 mg per patient per day for the statins or 1–2 g per patient per day for niclosamide. Tumor growth and metastasis formation to the liver of HCT116 cell was monitored over time using the ectopically overexpressed luciferase protein as described earlier) [17]. At the ethical end point, the animals were sacrificed, and the livers (site of distant metastasis) were snap-frozen in liquid nitrogen. To isolate tissue DNA from the livers including metastasis from human cells the liver tissue was randomly sliced using a cryomicrotome (Thermo Scientific, Waltham MA, USA). The amount of human satellite DNA in the liver tissue was detected by qPCR and immunohistochemistry (IHC) for human CK19, as previously described [25, 63].

### Statistical analysis

Data were analyzed using GraphPad Prism 8 (GraphPad Software Inc., San Diego CA, USA) and SPSS Statistics 21 (IBM, Armonk NY, USA). To compare datasets, Student’s *t*-tests (2 groups) and one-way ANOVA followed by correction for multiple comparison (Dunnett or Tukey, when comparing more than 2 groups) were employed. Statistical significance was assumed at a *p*-value < 0.05. Receiver operating characteristics (ROC) analysis was performed on mRNA expression levels to assess sensitivity and specificity for the primary endpoints “metachronous metastasis” and “death”. Youden’s J statistics were carried out to determine the optimal cut-off value to separate low and high MACC1 and S100A4 expression levels for subsequent survival analyses using Kaplan–Meier curves and logrank tests. Synergism of drug combinations on wound healing was analyzed at the 48 h time point using Combenefit v2.02 [64]. Bar graphs show mean and standard error of mean SEM.

## Supplementary information

Supplementary Movies

Supplementary Materials and Methods, Supplementary Figures

## Data Availability

The external CRC datasets [37, 38] are available via the Gene Expression Omnibus (https://www.ncbi.nlm.nih.gov/gds, accession number GSE28702 and GSE21510, respectively). Any other data used to support the findings of this study are available from the corresponding author on request.
