# Supplementary material for: Combinatorial treatment with statins and niclosamide prevents CRC dissemination by unhinging the MACC1-β-catenin-S100A4 axis of metastasis
Source: Oncogene. 2022 Aug 25;41(39):4446–58. doi: 10.1038/s41388-022-02407-6 (PMC9507965; doi:10.1038/s41388-022-02407-6)
Supplement: Supplementary file 1 — Supplementary Movies [file 41388_2022_2407_MOESM1_ESM.pptx]

## Slide 1
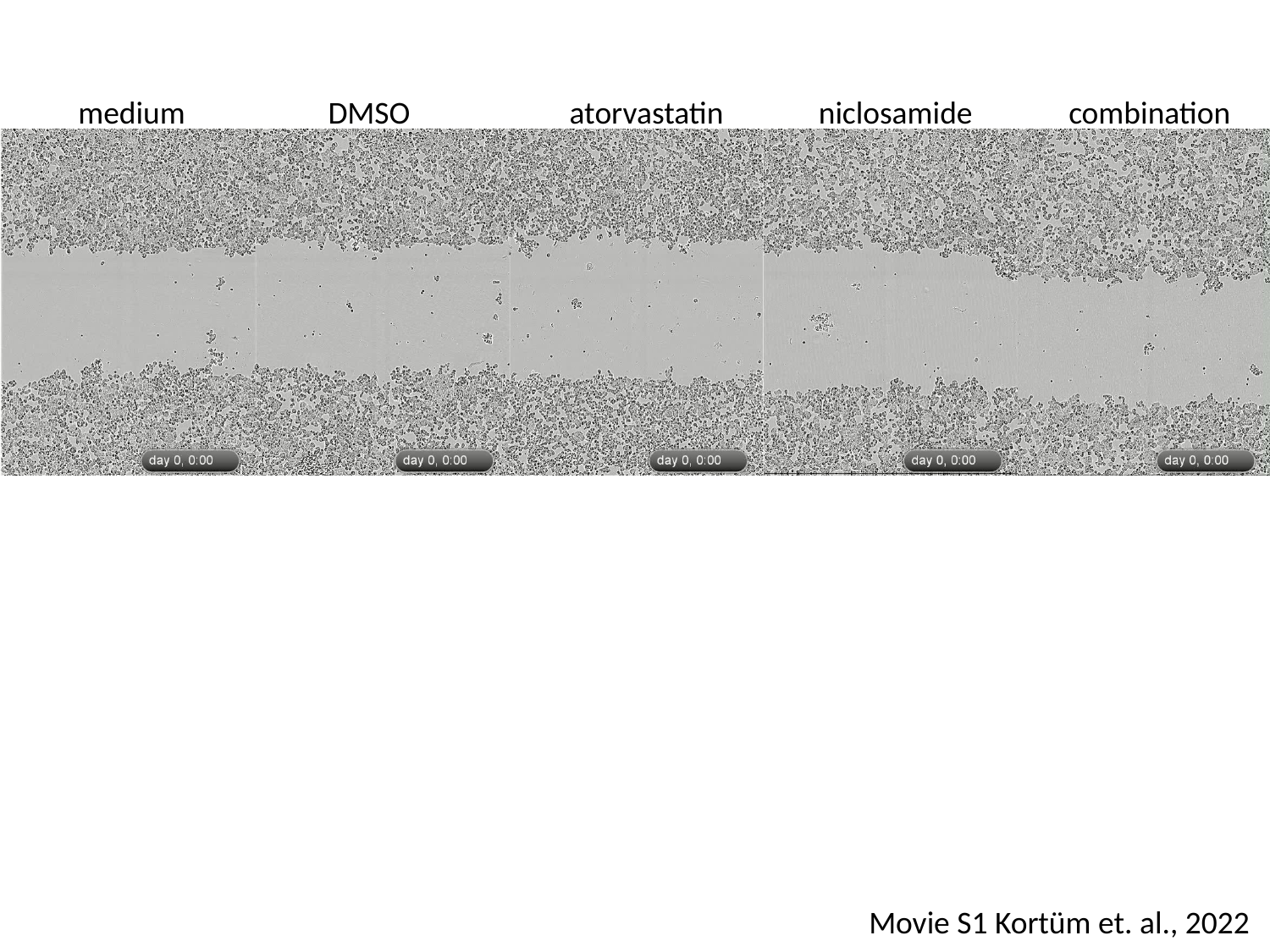

medium
DMSO
atorvastatin
niclosamide
combination
Movie S1 Kortüm et. al., 2022

## Slide 2
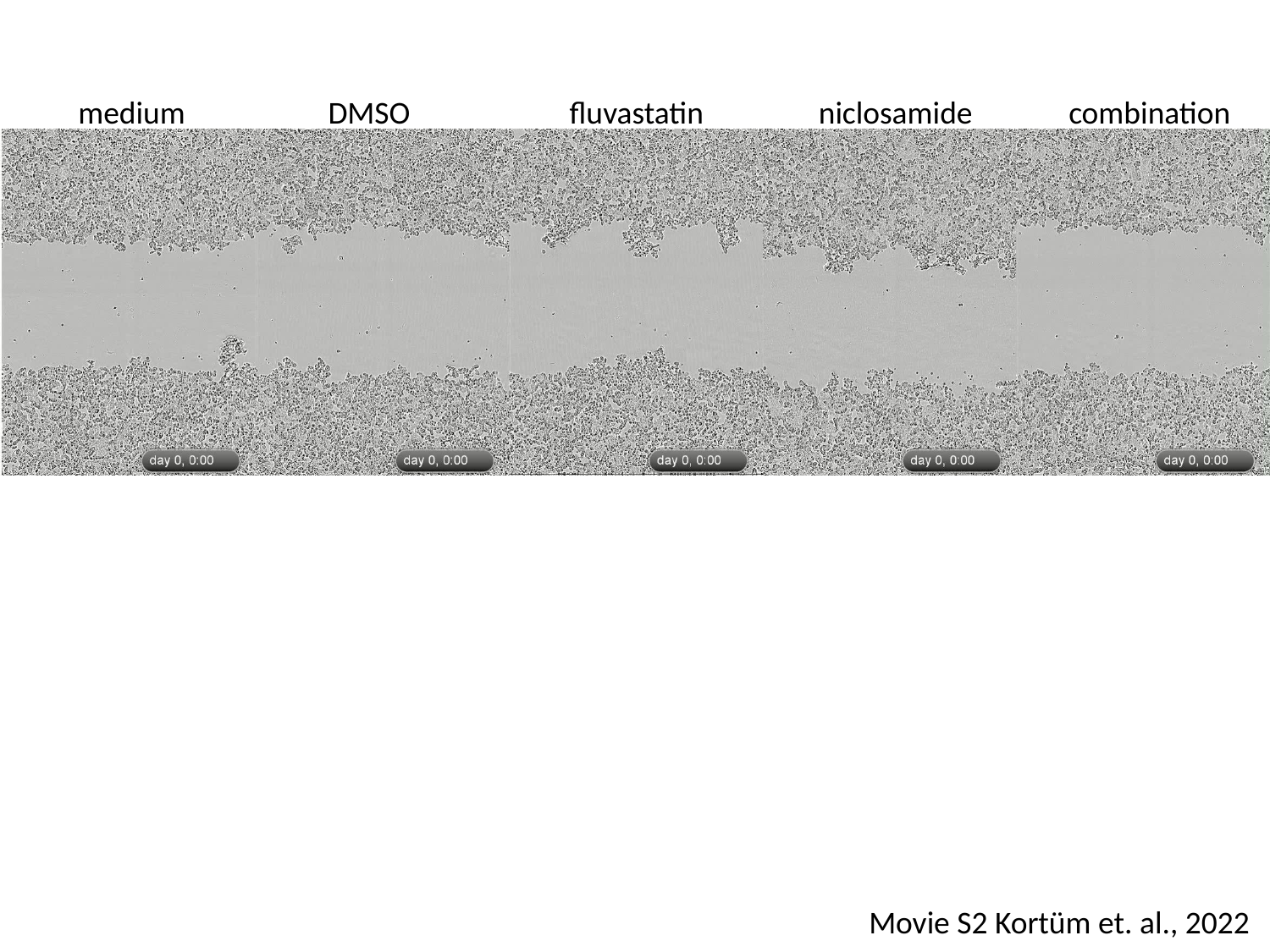

medium
DMSO
fluvastatin
niclosamide
combination
Movie S2 Kortüm et. al., 2022

## Slide 3
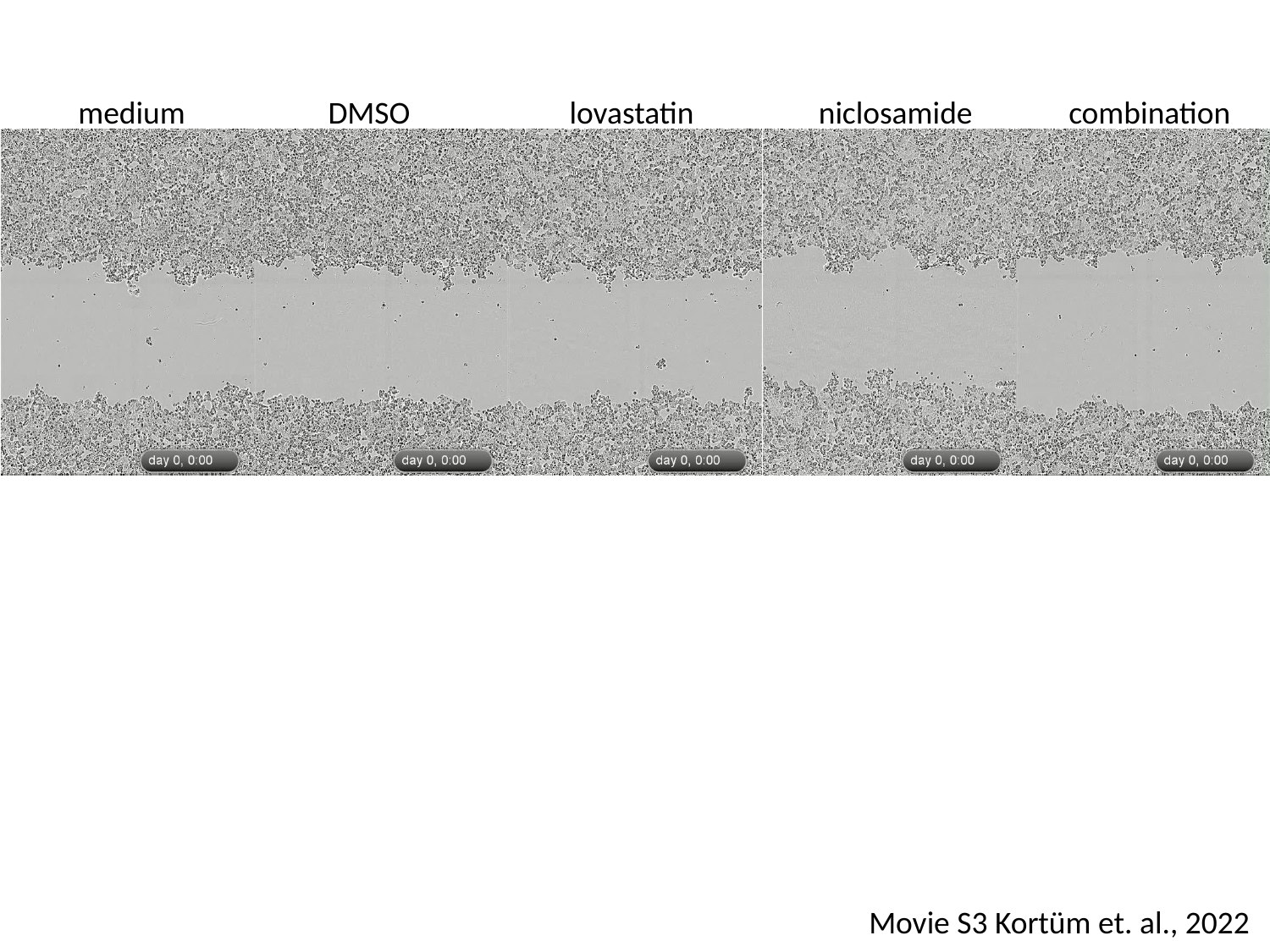

medium
DMSO
lovastatin
niclosamide
combination
Movie S3 Kortüm et. al., 2022
