# Supplementary material for: Combinatorial treatment with statins and niclosamide prevents CRC dissemination by unhinging the MACC1-β-catenin-S100A4 axis of metastasis
Source: Oncogene. 2022 Aug 25;41(39):4446–58. doi: 10.1038/s41388-022-02407-6 (PMC9507965; doi:10.1038/s41388-022-02407-6)
Supplement: Supplementary file 2 — Supplementary Materials and Methods, Supplementary Figures [file 41388_2022_2407_MOESM2_ESM.pdf]

Combinatorial treatment with statins and niclosamide prevents CRC dissemination by unhinging the MACC1-β-catenin-S100A4 axis of metastasis

Supplementary Materials

Contents:

Table S1-2

Figure Legends S1-S5

Supplementary Materials and Methods

Table S1:

sgRNA and knockout-sequencing primer		
sgMACC1	forward	5'- CAC ATC AAG TTC ATC ACC GGA GG -3'
sgS100A4	forward	5'- TTT GCC CGA GTA CTT GTG GAT GG -3'
MACC1 sequencing	forward	5'- GTA ACT CAC AGT GCC ACC TT -'3
	reverse	5'- AGC CAC TCT AAG TCG TGT AGT -'3
S100A4 sequencing	forward	5'- GAA TCT CCA GAG CTT GCG C -'3
	reverse	5'- AGC CAC CCC ACT GAT AGA TG -'3

Table S1: Summary of gRNAs for MACC1 and S100A4 knock out experiments and primers for sequencing.

Table S2:

Primers for qRT-PCR and sequencing		
hMACC1	forward	5'- TTC TTT TGA TTC CTC CGG TG-3'
	reverse	5'- ACT CTG ATG GGC ATG TGC TG -3'
hS100A4	forward	5'- CTC AGC GCT TCT TCT TTC -3'
	reverse	5'- GGG TCA GCA GCT CCT TTA -3'
mS100A4	forward	5'- TGA GCA ACT TGG ACA GCA ACA -3'
	reverse	5'- CTT CTT CCG GGG CTC CTT ATC -3'
mGapdh	forward	5'- AAC CTG CCA AGT ATG ATG AC -3'
	reverse	5'- CTG TTG CTG TAG CCG TAT T -3'
hRP-II	forward	5'- GAA GAT GGT GAT GGG ATT TC -3'
	reverse	5'- GAA GGT GAA GGT CGG AGT -3'
hCyclin-D1	forward	5'- CTG TTT GGC GTT TCC CAG AGT CAT C -3'
	reverse	5'- AGC CTC CTC CTC ACA CCT CCT C -3'
hMMP7	forward	5'- TCG GAG GAG ATG CTC ACT TCG A -'3
	reverse	5'- GGA TCA GAG GAA TGT CCC ATA CC -'3
hβ-catenin	forward	5'- GTG CTA TCT GTC TGC TCT AGT A -'3
	reverse	5'- CTT CCT GTT TAG TTG CAG CAT C -'3

Table S2: Summary of primers used for qPCR and sequencing.

Figure S1: Inhibition of S100A4 restricts MACC1-induced migration.

MACC1 induced cell migration in SW480 cells, and this effect was reverted by PCP, a structural inhibitor of S100A4 (A). MACC1 increased S100A4 in presence of DMSO, and niclosamide suppressed S100A4 on mRNA level equally in SW480/vector and SW480/MACC1 cells (B).

Figure S2: CRISPR-Cas9 mediated knockout of MACC1 and S100A4.

Knockout of MACC1 in SW620 and S100A4 in HCT116 was confirmed by gDNA isolation and targeted amplification of the region targeted by the single-guide RNA for Sanger sequencing. The sequence results were compared to the wildtype sequence of MACC1 or S100A4 using BLAST (https://blast.ncbi.nlm.nih.gov/Blast.cgi). The SW620 MACC1-KO clone used for all studies showed a major deletion of 141 bp, concomitant with loss of MACC1 expression on protein level. The HCT116 S100A4-KO clone used for cell motility assays showed a single nucleotide deletion, concomitant with loss of S100A4 expression on protein level.

Figure S3: Statins and niclosamide synergise in suppression of CRC cell wound healing.

HCT116 were treated with three concentrations (1.25, 2.5 and 5 μM) of fluvastatin (A) or lovastatin (B), and niclosamide (0.25, 0.5 and 1 μM) alone and in combinations thereof. After drug application *in vitro* wound healing was monitored for 48 h using the IncuCyte live cell imaging system. All statins and niclosamide were able to reduce wound closure compared to control cells. Combining a statin with niclosamide increased this effect synergistically. Results are shown as means ± SEM of at least 3 experiments.

Figure S4: Dosage optimization to maximise statin-niclosamide synergy for suppression of CRC metastasis.

Luminescence of metastases in murine livers following CRC cell xenografts under treatment with 100 % (A) and 12.5 % (B) human equivalent dosage of respective drug. Under 100 % dosage, all drugs significantly reduced liver metastases, and synergistic effects were obscured, while at 12.5 % no effects were observed, and dosage was not sufficient to elicit synergism of niclosamide and statin. Body weight curves normalised to control treated mice showed no toxicity of either treatment (C). Luminescence over time in the final group (D). IHC against hCK19 detected disseminated human CRC cells in murine liver (E) Representative murine liver sections show abundance of hCK19, indicating micrometastases (0 = healthy liver, 1 = solvent, 2 = niclosamide, 3 = fluvastatin, 4 = atorvastatin, 5 = fluvastatin + niclosamide, 6 = atorvastatin + niclosamide).

Figure S5: Restriction of liver metastasis from intrasplenic CRC SW620 cell transplantation in mice by statins and niclosamide.

The human CRC cell line SW620 with high endogenous MACC1 and S100A4 gene expression was xenografted into the spleen of SCID beige mice. Treating the mice (n = 60, 10 animals per group) with human equivalent doses of either atorvastatin (13 mg/kg, 80 mg per patient per day), fluvastatin (13 mg/kg, 80 mg per patient per day) or niclosamide (328 mg/day, 2 g per patient per day) alone did not reduce metastasis formation in the liver. Confirming the *in vitro* and *in vivo* results achieved with cell line HCT116, the combination of the tested drugs, each statin with niclosamide, was superior to single drug treatment to reduce metastasis formation to the liver. Metastases load was quantified at the experimental endpoint by human satellite DNA qPCR (A) and human CK19 staining of liver tissue (B) to detect human CRC cells in murine liver tissue. Scale bars represent 100, 50 or 25 μM for magnifications of 10x, 20x, and 40x, respectively.

Supplementary materials and methods

Mass-spectrometry based proteomics

The collected proteins were the digested according to Kanashova et al. 2015[1]. The purified peptides were measured on a Q-Exactive plus mass spectrometer (Thermo-Fisher) coupled to a Proxeon nano-LC system (Thermo-Fisher) in data-dependent acquisition mode, selecting the top 10 peaks for HCD fragmentation. A 3-h gradient (solvent A: 5 % acetonitrile, 0.1 % formic acid; solvent B: 80 % acetonitrile, 0.1 % formic acid) was applied for the samples using an in-house prepared nano-LC column (0.075 mm × 250 mm, 3 µm Reprosil C_{18}, Dr. Maisch GmbH). A volume of 5 µl sample was injected and the peptides eluted with 3 h gradients of 4 to 76 % ACN and 0.1 % formic acid in water at flow rates of 0.25 µl/min. MS acquisition was performed at a resolution of 70,000 in the scan range from 300 to 1700 m/z. Dynamic exclusion was set to 30 s and the normalised collision energy to 26 eV. The mass window for precursor ion selection was set to 2.0 m/z. The MS2 ion count target was set to 2×10^3 and the max injection time was 300 ms. Only precursors with a charge state of 2–7 were sampled for MS2. The collected data was interpreted using the MaxQuant software suite (version 1.5.2.8)[2] using the human UniProt database (downloaded 06.08.2014)[3] with carbamylation of cysteins set as a fixed modification and oxidation of methionines and N-terminal protein acetylation set as variable modifications[2,4]. The statistical analysis was performed using the R-software[5].

TOP-Flash and promoter reporter assays

To measure β-catenin/TCF4 pathway activity, 7.5 × 10^4 cells were seeded a day before transfection with 500 ng of plasmids encoding TOP-Flash or FOP-Flash [6] upstream of firefly luciferase using Lipofectamine 2000 (Thermo Fisher). 25 ng renilla luciferase (stable expression under a CMV promoter) served as transfection control. Firefly and renilla luciferase activity were measured with the Dual-Luciferase Assay (Promega) according to the manufacturer's instructions. S100A4 promoter activity was measured with an analogue protocol, but with the S100A4 promoter upstream of firefly luciferase [7].

qRT-PCR

RNA transcripts were quantified in quantitative RT-PCR (qRT-PCR) assays. Briefly, RNA of 2 × 10^5 cells treated as indicated for 24 h was isolated with the GeneMATRIX universal RNA purification kit (EURx, Gdansk, Poland). After quantification (Nanodrop, Peqlab, Erlangen, Germany), 50 ng of RNA was reverse transcribed with random hexamers in a reaction mix (10 mM $MgCl_2$, 1 × RT Buffer, 250 µM pooled dNTPs, 1 U RNAse inhibitor, 2.5 U Moloney murine leukemia virus reverse transcriptase; all from Biozym) at 30°C for 10 min, 50°C for 40 min and 99°C for 5 min, followed by cooling at 4°C. PCR was carried out on a LightCycler 480 system (Roche) at 95°C for 2 min followed by 40 cycles of 95°C for 5 s and 60°C for 20 s using exon-spanning primers. Primer sequences are listed in Table S2.

Western blot, Co-immunoprecipitation

PBS-washed cells were lysed with RIPA buffer (50 mM Tris-HCl pH 7.5, 150 mM NaCl, 1 % Nonidet P-40) supplemented with cOmplete protease inhibitor cocktail and phosStop

phosphatase inhibitor (Roche), when indicated. After 30 min of lysis on ice and centrifugation at 12,000 × g for 10 min at 4°C, protein concentration was quantified with the Pierce BCA system (Thermo Fisher). 2 µg (for total protein analysis) or 30 µg (for phospho-protein analysis) of protein were boiled with 0.1 M DTT (Sigma) and 1 × LDS loading buffer (NuPage, Invitrogen). Cellular proteins were resolved in SDS page and transferred onto PVDF membranes, followed by blocking with 5 % w/v skim milk powder in TBS-T (10 mM Tris-HCl pH 8, 150 mM NaCl and 0.1 % Tween 20) for 1 h. Proteins of interest were detected by probing membranes with specific primary antibodies at 4°C overnight and secondary HRP-conjugated antibodies for 1 h at room temperature, and subsequent visualization with WesternBright (Advansta, Menlo Park, CA, USA) and exposure of Fuji medical X-ray film SuperRX (Fujifilm, Tokyo, Japan). For Co-immunoprecipitation (Co-IP) experiments, cells were grown to ~80 % confluence in 10 cm dishes, washed twice with ice-cold PBS and scraped off with 1 ml Co-IP buffer (20 mM Tris-HCl pH 7.5, 150 mM NaCl, 0,1 % Nonidet P-40, 1 mM EDTA, 1 % Triton X100) in the presence of protease and phosphatase inhibitors. Lysates were incubated with 1 µg specific antibody or normal immunoglobulin G under constant agitation overnight at 4°C, and addition of agarose beads (AlphaBiosciences) for another 4 h at 4°C. Protein-antibody complexes were precipitated by centrifugation at 2,500 × g for 5 min. The agarose pellets were washed 5 times by resuspending in CoIP buffer and centrifugation at 2,500 × g. To detect protein-protein interactions, the agarose beads were eluted by boiling in 2 × loading buffer, separation on SDS page and Western blot. The following primary antibodies were used: MACC1 (HPA020081, Sigma Aldrich), S100A4 (A5114, DAKO), β-catenin (610154, BD Biosciences), p-Ser-552 (9566, CST), TCF7L2 (ab76151,

abcam), β-actin (A1978, Sigma Aldrich). For supernatant WB, 1 × 10^6 cells were seeded into a 6-well plate, and complete medium was replaced with 1.5 ml serum-free culture medium. After 24 h, the cell culture supernatant was carefully collected and floating cells were eliminated by centrifugation at 500 × g for 5 min. a portion of clarified cell culture medium was boiled with 0.1 M DTT (Sigma) and 1 × LDS loading buffer (NuPage, Invitrogen). Meanwhile, cells from the same well were counted in order to load proportional medium samples into SDS-Page.

In silico correlation analyses of MACC1 and S100A4 expression in CRC

We obtained expression data of CRC tumour microarrays from the public functional genomics data repository Gene Expression Omnibus (www.ncbi.nlm.nih.gov/geo). 98 expression datasets (GDS4393 and GDS4718)[8,9] of MACC1 and S100A4 were analysed for their correlation. Similarly, MACC1 and S100A4 gene expression of 117 CRC tissue sample from the OncoTrack consortium[10] dataset were processed. Spearman correlation coefficients were calculated after normalising to G6PDH and illustrated as scatter plots.

References

1 Kanashova T, Popp O, Orasche J, Karg E, Harndorf H, Stengel B *et al.* Differential proteomic analysis of mouse macrophages exposed to adsorbate-loaded heavy fuel oil derived combustion particles using an automated sample-preparation workflow. *Anal Bioanal Chem* 2015; **407**: 5965–76.

2 Cox J, Mann M. MaxQuant enables high peptide identification rates, individualized p.p.b.-range mass accuracies and proteome-wide protein quantification. *Nat Biotechnol* 2008; **26**: 1367–72.

3 UniProt: a worldwide hub of protein knowledge. *Nucleic Acids Res* 2019; **47**: D506–D515.

4 Cox J, Neuhauser N, Michalski A, Scheltema RA, Olsen J V, Mann M. Andromeda: a peptide search engine integrated into the MaxQuant environment. *J Proteome Res* 2011; **10**: 1794–805.

5 3.5.1. RDCT. A Language and Environment for Statistical Computing. *R Found Stat Comput* 2018; **2**: https://www.R-project.org.

6 Korinek V, Barker N, Morin PJ, van Wichen D, de Weger R, Kinzler KW *et al.* Constitutive transcriptional activation by a beta-catenin-Tcf complex in APC-/- colon carcinoma. *Science* 1997; **275**: 1784–7.

7 Stein U, Arlt F, Walther W, Smith J, Waldman T, Harris ED *et al.* The Metastasis-Associated Gene S100A4 Is a Novel Target of ß-catenin/T-cell Factor Signaling in

Colon Cancer. *Gastroenterology* 2006; **131**: 1486–1500.

8 Tsuji S, Midorikawa Y, Takahashi T, Yagi K, Takayama T, Yoshida K *et al.* Potential responders to FOLFOX therapy for colorectal cancer by Random Forests analysis. *Br J Cancer* 2012; **106**: 126–32.

9 Tsukamoto S, Ishikawa T, Iida S, Ishiguro M, Mogushi K, Mizushima H *et al.* Clinical significance of osteoprotegerin expression in human colorectal cancer. *Clin Cancer Res* 2011; **17**: 2444–50.

10 Henderson D, Ogilvie LA, Hoyle N, Keilholz U, Lange B, Lehrach H *et al.* Personalized medicine approaches for colon cancer driven by genomics and systems biology: OncoTrack. *Biotechnol J* 2014; **9**: 1104–1114.

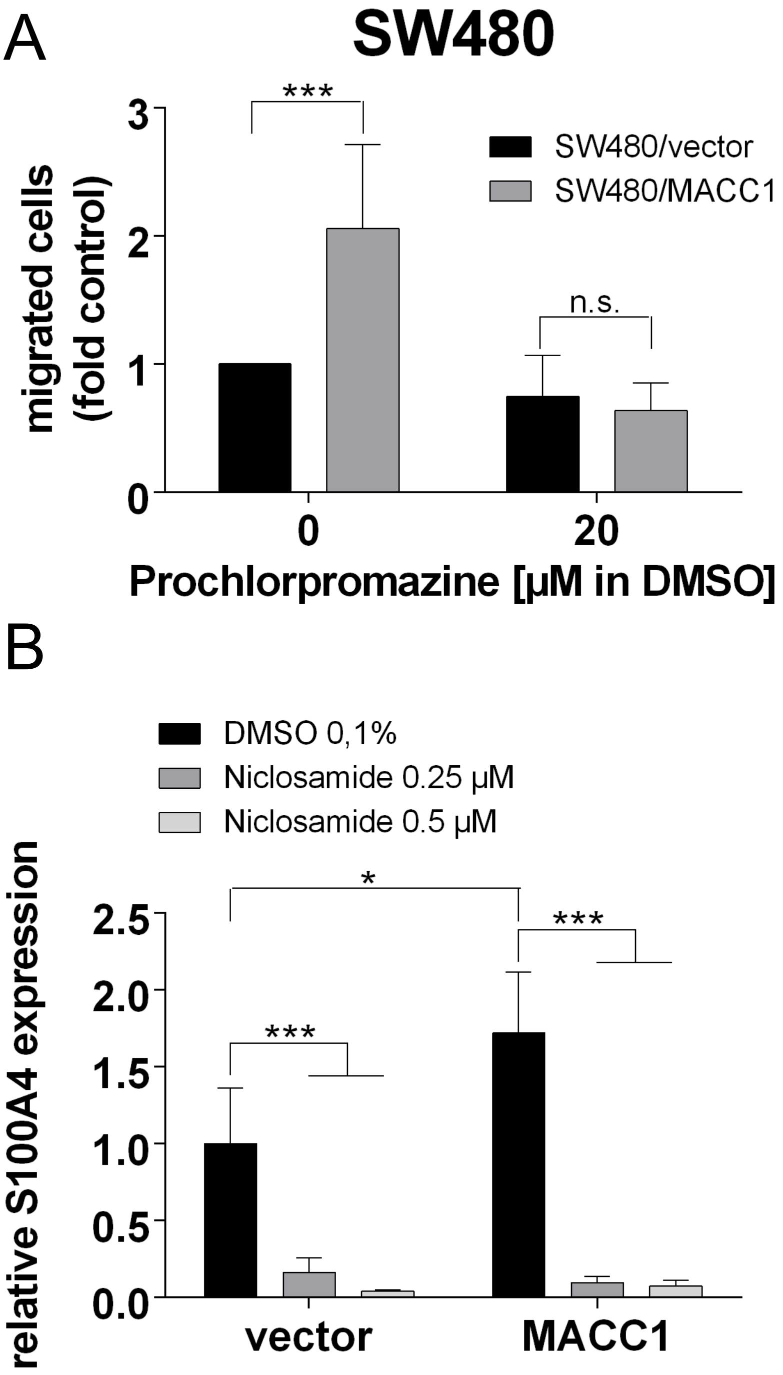

Fig. S1 Kortüm et al., 2022

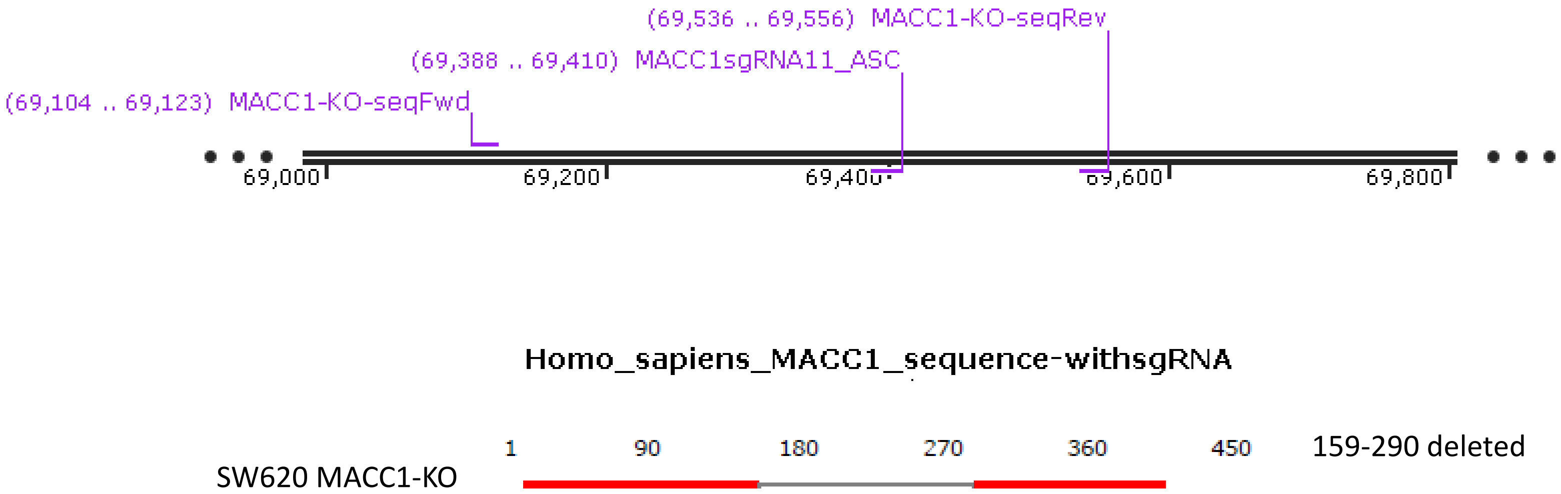

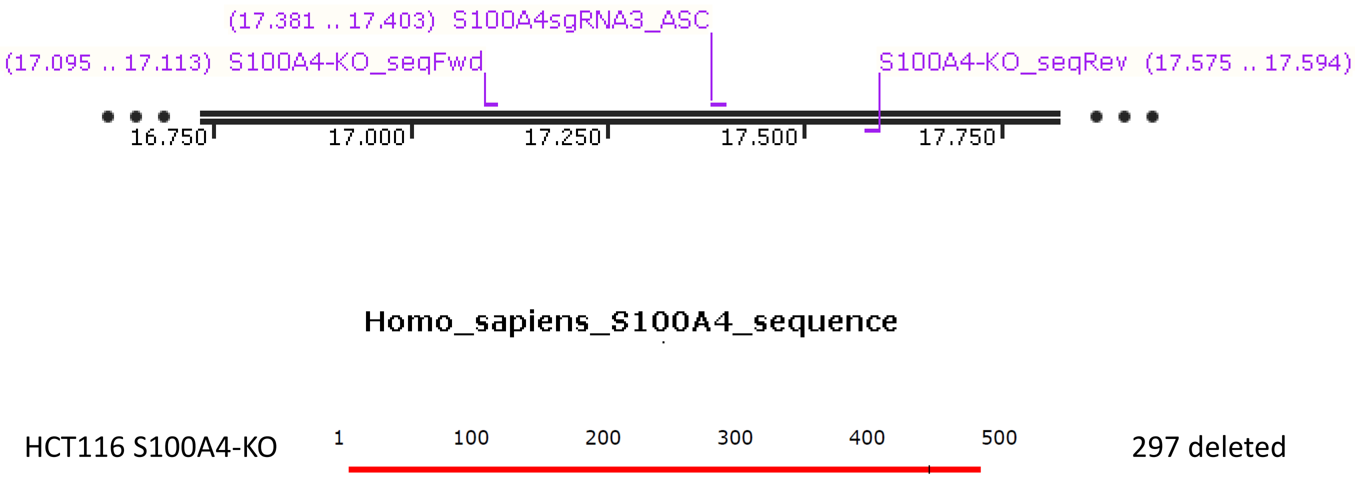

Fig. S2 Kortüm et al., 2022

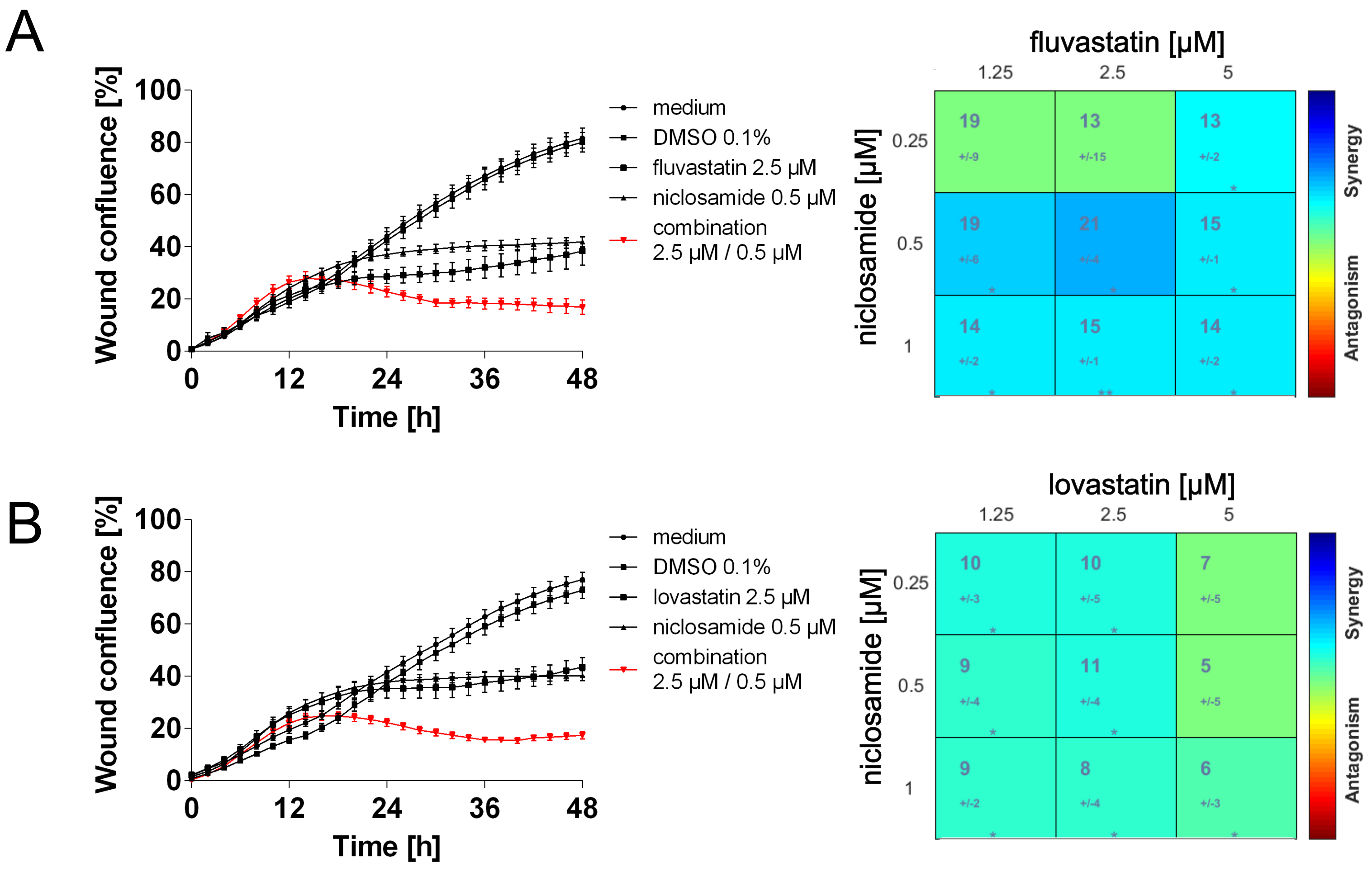

Fig. S3 Kortüm et al., 2022

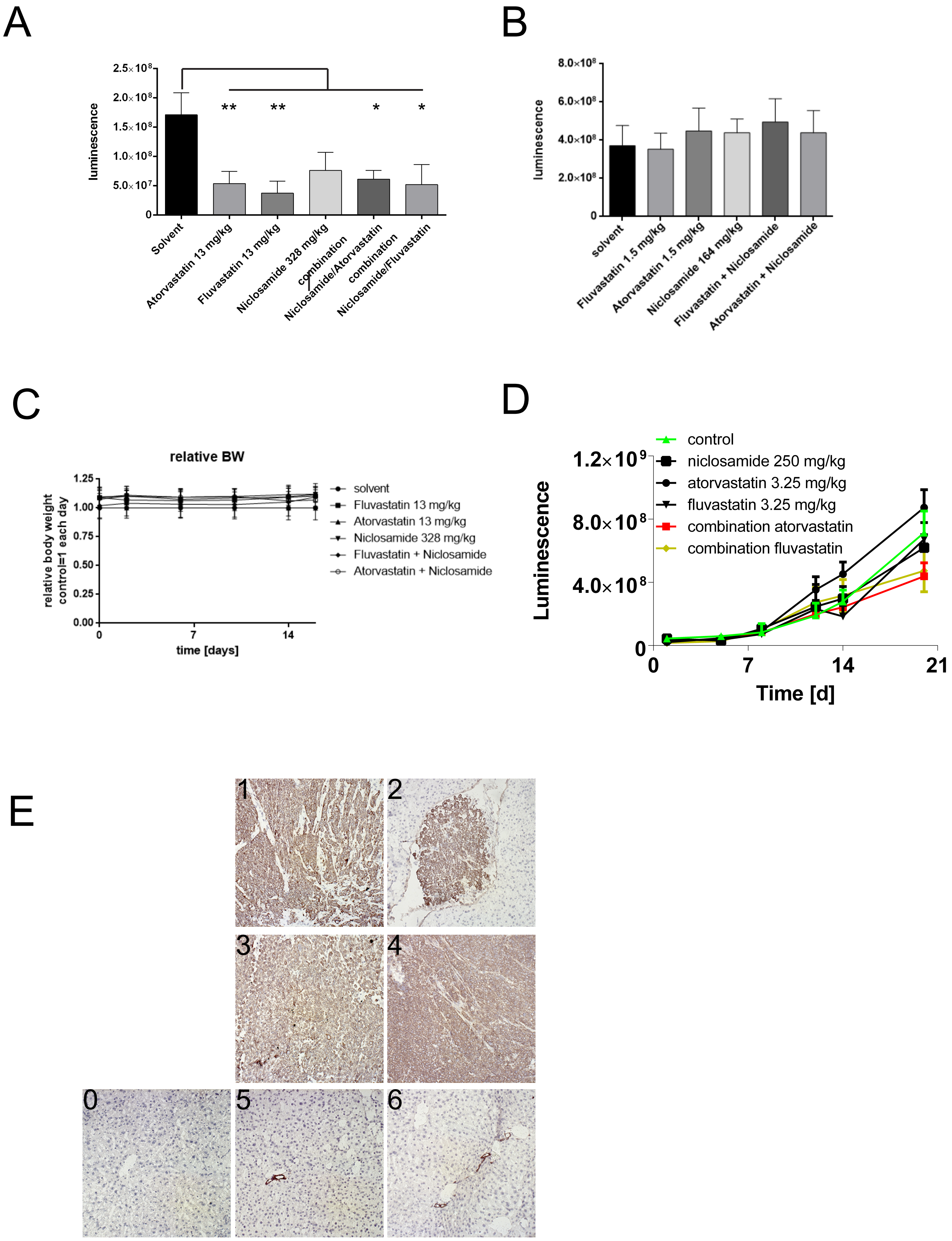

Fig. S4 Kortüm et al., 2022

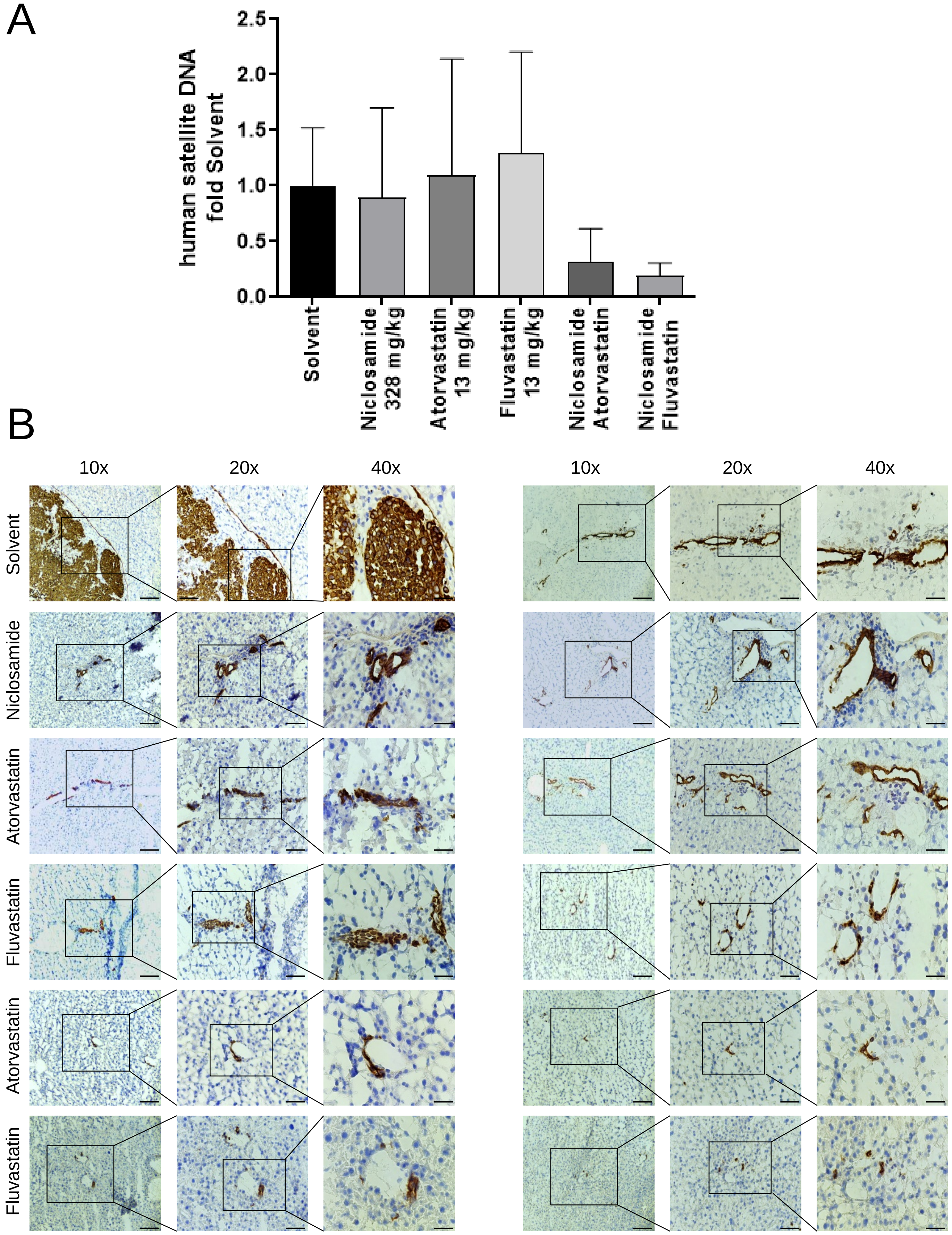

Fig. S5 Kortüm et al., 2022
